# Risk factors for cutaneous leishmaniasis in the rainforest of Bolivia: a cross-sectional study

**DOI:** 10.1186/s41182-018-0089-6

**Published:** 2018-04-17

**Authors:** Daniel Eid, Miguel Guzman-Rivero, Ernesto Rojas, Isabel Goicolea, Anna-Karin Hurtig, Daniel Illanes, Miguel San Sebastian

**Affiliations:** 1 Institute of Biomedical Research, Faculty of Medicine, San Simon University, Aniceto Arce Avenue 371, Cochabamba, Bolivia; 2 Department of Public Health and Clinical Medicine, Epidemiology and Global Health, Umea University, Umea, Sweden

**Keywords:** American cutaneous leishmaniasis, Risk factors, Epidemiology, Bolivia

## Abstract

**Background:**

Cutaneous leishmaniasis (CL) is an endemic disease in Bolivia, particularly in the rainforest of Cochabamba, in the municipality of Villa Tunari. The precarious, dispersed, and poorly accessible settlements in these farming communities make it difficult to study them, and there are no epidemiological studies in the area. The aim of the present study was to identify the risk factors associated with cutaneous leishmaniasis.

**Methods:**

A cross-sectional study was conducted in August 2015 and August 2016 in two communities of Villa Tunari, Cochabamba. The cases were diagnosed through clinical examinations, identification of the parasite by microscopic examination, and the Montenegro skin test. Risk factors were identified through logistic regression.

**Results:**

A total of 274 participants (40.9% female and 59.1% male) were surveyed, of which 43% were CL positive. Sex was the only factor associated with CL with three times more risk for men than for women; this finding suggests a sylvatic mechanism of transmission in the area.

**Conclusions:**

It is advisable to focus on education and prevention policies at an early age for activities related to either leisure or work. Further research is needed to assess the influence of gender-associated behavior for the risk of cutaneous leishmaniasis.

## Background

American cutaneous leishmaniasis (ACL) is an infectious disease produced by the parasite Leishmania spp. This is transmitted by the bite of sandflies that carry the parasite from reservoirs to humans. Reservoirs for the parasite are mostly rodents and large wild mammals. Humans are accidental hosts when they invade the reservoirs and vectors’ ecosystem [1]. In humans, ACL is characterized by chronic skin ulcers that can take from months to years to heal [2].

In 2008, the estimated annual incidence of cutaneous leishmaniasis (CL) in America reached up to 307,800 new cases [3] and it is estimated that 39 million of people are at risk in 21 countries from the Caribbean to South America [4]. The Amazon rainforest is an especially risky area because it is the habitat of the reservoirs and vectors of the Leishmania parasite. This region includes nine nations, and Bolivia is the third country with the most amount of forest, which makes up 70% of its territory. National reports estimate 2300 new cases every year; however, this could be higher due to under-reporting [5]. Incidence rates per 100,000 inhabitants have increased four times during the past 35 years from 4.8 in 1983 to 18.5 in 2012 [6]. This situation can be explained by the accelerated process of deforestation, migration, and colonization of the Bolivian forests for agriculture and for the precariousness and poverty of the settlements. Although these social phenomena allow us to explain the populations’ risk, they do not explain the characteristics at the individual level that define the risk to these exposed populations of becoming ill.

Over the past two decades, several studies have addressed the question of risk factors for ACL. One frequent factor is the human settlements close to a primary forest. When the ecological environments are disturbed, humans are more likely to be exposed to reservoirs and vectors increasing the risk for ACL [7]. Other common factors include gender, age, and outdoor activities. There is a pattern that in sylvatic contexts, men of working age are usually more exposed because of their activities in agriculture and forestry [8–13].

Another group of factors are related to housing conditions. Studies have shown that when walls, roofs, and floors are not made of durable materials, cracks can be formed, becoming a shelter or a gateway for vectors into the households [11]. Some studies have also found that using wood as a cooking fuel was a risk factor arguing that it would increase exposure when it is used in open environments [11]. Other studies have however identified this as protective arguing that probably the smoke drives away the vectors [9].

Indoor electricity use has also produced contradictory results. In a study carried out in Ecuador, this turned out to be protective and the authors proposed that light attracts vectors and distances them from people [9]. However, other studies have not confirmed these findings [13].

Another contradictory factor is the presence of domestic animals such as dogs, pigs, or chickens. Some studies have found a protective role in their presence arguing that animals were a preferred source of blood for vectors [10, 14]; however, other studies have suggested that animals could attract the vectors closer to humans, thus becoming an important risk factor [13, 15, 16].

The only study in Bolivia about risk factors was carried out 20 years ago, and the main risk factors reported were occupation, age, and migration [8]; however, this study has only focused on well-known risk factors involved in sylvatic chains of transmission. The constant process of migration, deforestation, and colonization of the forest for agriculture creates the conditions needed to change the leishmaniasis transmission profile from an occupational disease in the forest to a domestic disease in the communities [17]. The purpose of this paper was to assess sociodemographic and housing condition factors as risk factors for CL in a rural area of the Bolivian Amazon.

## Methods

### Study area

**Map 1:**
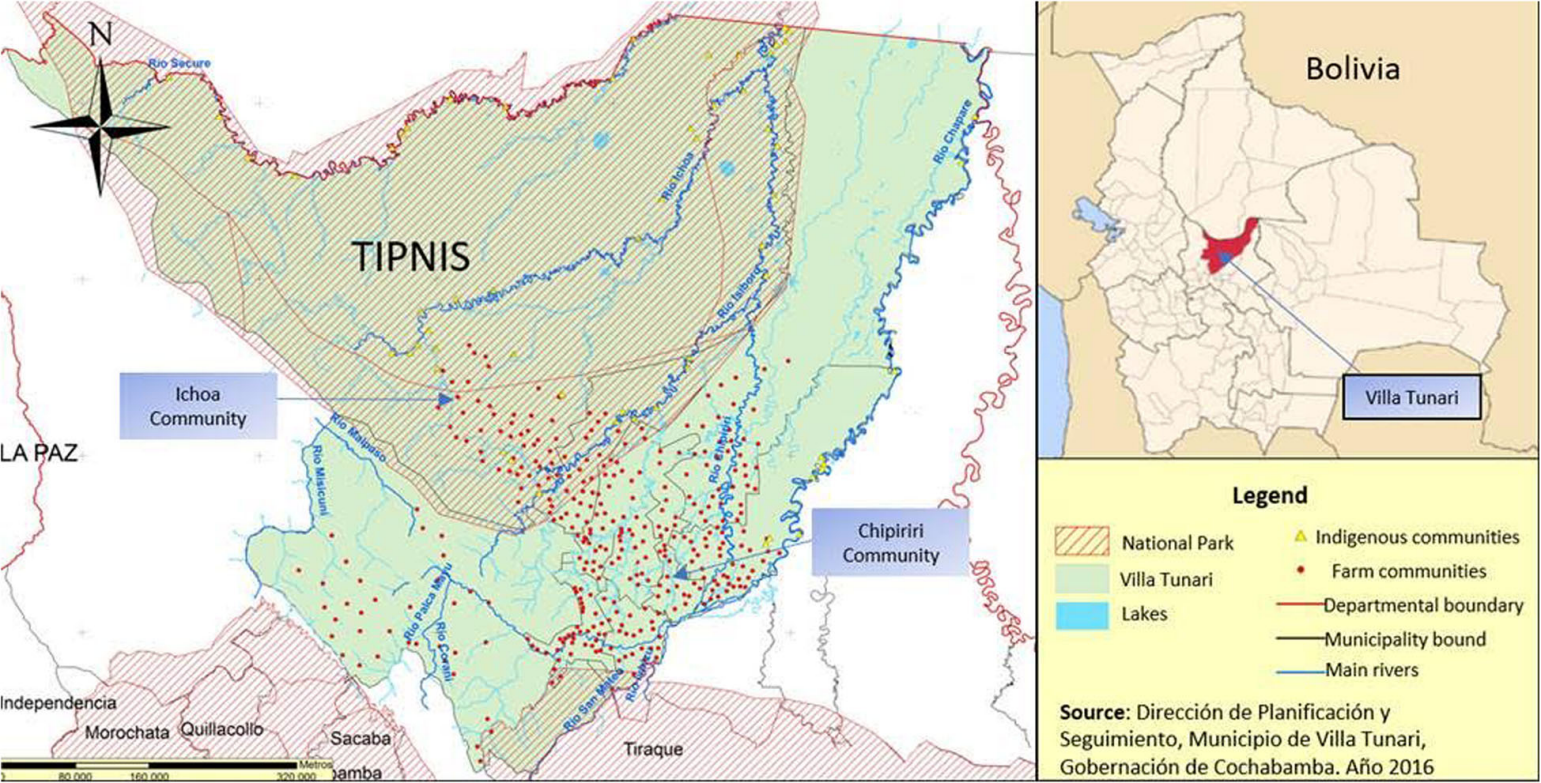
Map of Villa Tunari Municipality showing distribution of communities and the ecological reserve of TIPNIS

The study was conducted in the municipality of Villa Tunari located in the tropical forest of Cochabamba department (Map 1). In addition, this municipality contains one of the highest ecological reserves in the country, the Isiboro Secure National Park and Indigenous Territory (TIPNIS).

The population registered during the national census in 2012 was 73,914 inhabitants. Migrants represent around 44% of the population, mostly coming from the occidental highlands. The colonization of the tropical forest for agricultural activities is an ongoing process which has been highly criticized by its demographically and environmentally destructive nature [18]. From 1992 to 2012, the population increased by 52%, and today, more than 400 communities are registered in the municipality, 90 of them located in the TIPNIS. These migration movements have also had an impact on the epidemiological profile of leishmaniasis. More than 60% of CL cases in the tropical zone of Cochabamba come from this area, making it one of the municipalities with the highest risk of CL in the country [6].

These human settlements are commonly created in poverty-stricken conditions. The National Census in 2012 reported that nearly all the population in Villa Tunari municipality lived below the poverty line (95%), and many in extreme poverty (22%) in terms of unsatisfactory basic living conditions [19]. Water supply depends on rivers or ponds for most of the households (81%), and firewood is the main cooking fuel in the houses (62%). More than half of the households do not have electricity (56%), and only a few have a sewer system (12%).

Villa Tunari municipality is organized in towns and villages. The primary forest is mainly located in the TIPNIS while outside of the national reserve is mostly secondary forest. Two villages were selected for this study representing different levels of urbanization and vector exposure. Ichoa is located close to the primary forest inside the TIPNIS, and Chipiriri is located outside the reserve representing the secondary forest.

### Data collection

A cross-sectional study was conducted during visits to the communities of Ichoa in August 2015 and Chipiriri in August 2016 spending 6 days in each community. The population living in these communities was invited to participate in a CL and ML screening-based study. Multiple invitations were made by their local authorities, by the medical staff of the health center, and by the researchers during their community meetings. The strategy of searching cases trough medical campaigns was chosen instead of house-to-house visits because it was not feasible to find people in their houses. People used to be working on their farms during the day far from their households. In addition, the sylvatic geography where they are settled as well as the scattered distribution of the households made it complicated to find the participants at night.

Medical campaigns consisted of comprehensive physical examination of skin and mucous membranes focused on the detection of present or past cutaneous leishmaniasis conducted by a group of senior researchers from the Center of Tropical Medicine of San Simon University in Cochabamba. Whenever a skin ulcer was present in the participants, a sample was taken for direct microscopic examination (DME).

The criteria used to identify CL infections were (1) presence of skin ulcers on which Leishmania parasite was identified through DME (sensitivity of 50–68%) [20, 21] and (2) skin scars with history of skin ulcers that lasted more than 2 weeks before healing and who would had a positive reaction to the Montenegro skin test (MST). This test has been successfully used in several epidemiological studies to assess exposition to the Leishmania parasite and leishmaniasis infection [22–27]. The MST sensitivity and specificity to detect parasite exposure were reported at over 97 and 93%, respectively, which improves with increased exposure time [28–30]. The reaction to MST was measured after 48 h by the ballpoint pen method; if induration was 5 mm or larger, the test was considered positive. The antigens used for the MST test were produced by the Institute of Tropical Medicine at the Cayetano Heredia University, Peru.

A questionnaire was used to collect data regarding house construction materials (wall and roof), basic services (electricity, gas, drinking water networks, and sewer system), and sociodemographic characteristics (age, gender, farming activity, schooling level, and mother tongue) which was adapted from the Demography and Health National Survey [31]. Finally, information related to forest exposure and use of protective measures against mosquito bites such as the use of bed nets, use of repellants, and household fumigations were collected from all the participants.

### Statistical analysis

The descriptive statistics were expressed as percentages and as a mean and standard deviation (SD) for categorical and continuous variables, respectively. Logistic regression was used to test the association between the potential risk factors and the outcome. A univariate analysis was performed with all the variables included in the study, and those with a *p* value less than 0.2 were included in the multivariate analysis. There was no collinearity among the variables included in the multivariate analysis assessed with the variance inflation factor. All analyses were carried out using the SPSS software.

## Results

The sample consisted of 274 subjects from Ichoa community (*n* = 200) and Chipiriri community (*n* = 74). Participants were mostly young people (median age 35 years old). Work activities were mostly agriculture for men (79.6%) and housework for women (78.6%). Migrant people from the highlands represented more than half of the sample (56.6%), and their mother tongue was mostly Quechua (82.8%). The educational level was 6.2 years on average. Of the total sample, 119 participants (43.4%) had CL, and of them, 116 cases (97.5%) corresponded to past infections with skin scars and history of chronic infection that were positive for MST, while 3 cases (2.5%) corresponded to active skin ulcers that were positive for DME. The majority of CL cases were men (69.7%), and the median age was 35 years old (range 14–73). Most cases were from the Ichoa community (79.8%) compared to the Chipiriri community (20.2%). A greater proportion of cases were migrants from the highlands (58.0%), with Quechua and Aymara as their mother tongue (87.4%) and with a residence time in the communities greater than 10 years (54.2%). More than half of the cases worked in agriculture (64.7%), and although the majority of the cases could read (89.0%), the schooling level was low (6.2 years of education in average) (Table 1).

**Table 1:** Sociodemographic characteristics of the participants expressed in percentages, Villa Tunari 2015

Variable		*N* (%)	CL positive *N* (%)
Sex	Female	112 (40.8)	36 (30.3)
	Male	162 (59.1)	83 (69.7)
Age in years	Median (min–max)	35 (12–73)	35 (14–73)
Residence	Chipiriri community	74 (27.0)	24 (20.2)
	Ichoa community	200 (73.0)	95 (79.8)
Years of residence	Below 10	136 (49.8)	54 (45.8)
	More than 10	137 (50.2)	64 (54.2)
Migrant from highlands	No	119 (43.4)	50 (42.0)
	Yes	155 (56.6)	69 (58.0)
Mother tongue	Spanish	35 (12.8)	15 (12.6)
	Quechua or Aymara	239 (87.2)	104 (87.4)
Farmer	No	121 (44.2)	42 (35.3)
	Yes	153 (55.8)	77 (64.7)
Reading	No	38 (13.9)	13 (11.0)
	Yes	235 (86.1)	105 (89.0)
Education in years	Mean (SD)	6.2 (3.7)	6.3 (3.6)

Regarding the housing conditions of the sample, wall materials were mostly wood or plastered mud (70.7%), the drainage system was mostly cesspool or none (81.7%), and the fuel used for cooking was primarily firewood (77.6%). Houses were mostly small having fewer than three bedrooms (64.2%), and the human density was 2.7 people per bedroom on average. Most of the participants raised hens (86.5%), while less than half owned dogs (40.4%). Housing conditions of cases showed some interesting findings. There was a greater proportion of cases with wood (78.2%) compared to concrete (21.8%) as the material of the walls, with cesspool or none (88.2%) as the drainage system, and wood (81.4%) instead of gas (18.6%) as the cooking fuel. Additionally, the majority of cases lived in households with less than three rooms (71.4%). Finally, there were more cases who had hens (92.0%) but less of those who owned dogs (38.6%) (Table 2).

**Table 2:** Housing conditions of the participants expressed in percentages, Villa Tunari 2015

Variable		*N* (%)	CL positive *N* (%)
Wall material	Brick or concrete	80 (29.3)	26 (21.8)
	Wood and others	193 (70.7)	93 (78.2)
Drainage system	Septic tank	50 (18.3)	14 (11.8)
	Cesspool or none	223 (81.7)	105 (88.2)
Cooking fuel	Wood	209 (77.1)	96 (81.4)
	Gas	62 (22.9)	22 (18.6)
Number of rooms	Less than 3	176 (64.2)	85 (71.4)
	3 or more	98 (35.8)	34 (28.6)
Persons per room	Mean (SD)	2.7 (1.7)	2.9 (1.8)
Dog ownership	No	134 (59.6)	62 (61.4)
	Yes	91 (40.4)	39 (38.6)
Hens ownership	No	30 (13.5)	8 (8.0)
	Yes	193 (86.5)	92 (92.0)

Regarding the use of protective measures of the sample, the routine use of repellent was rare (2.9%), and the majority described having sprayed the house sometimes or very often (79.5%). Almost all the participants used a bed net to sleep (90.5%). Most participants described sleeping with open windows (68.6%). Among CL cases, almost all referred to always use bed nets (92.4%); however, most of them always slept with the windows open (66.7%). The majority of the cases fumigated their household sometimes (54.2%) or always (23.7%) and never used repellents (78.0%) (Table 3).

**Table 3:** Prevention activities of the participants expressed in percentages, Villa Tunari 2015

Variable		*N* (%)	CL positive *N* (%)
Use of bed nets	Some times	26 (9.5)	9 (7.6)
	Always	247 (90.5)	109 (92.4)
Use of repellent	Never	204 (74,7)	92 (78.0)
	Some times	61 (22,3)	25 (21.2)
	Always	8 (2,9)	1 (0.8)
Household fumigations	Never	56 (20,5)	26 (22.0)
	Some times	145 (53,1)	64 (54.2)
	Very often or always	72 (26,4)	28 (23.7)
Sleeping with windows open	Some times	26 (31,4)	35 (33.3)
	Always	247 (68,6)	70 (66.7)

In the univariate analysis, the sociodemographic factors associated with an increased risk of CL were to be male, a farmer, and living in a village close to the primary forest. Some housing conditions such as wall materials, sewage system, number of rooms in the house or number of persons per room, and ownership of chicken were also significant. In the multivariate analysis, sex remained as the only one statistically significant risk factor where men had three times increased odds of being infected with CL compared to women (Table 4).

**Table 4:** Univariate and multivariate analysis of sociodemographic, housing conditions, and protective activities associated with CL

Factor		Unadjusted OR with 95%CI	Adjusted OR with 95%CI
Sex	Female	1.0	1.0
	Male	2.2 (1.3–3.7)	3.2 (1.6–6.6)
Age in years	Mean (SD)	1.0 (1.0–1.0)	
Residence	Chipiriri community	1.0	1.0
	Ichoa community	1.9 (1.1–3.3)	1.9 (0.6–5.7)
Years of residence	More than 10	1.0	
	Below 10	1.3 (0.8–2.2)	
Migrant from highlands	No	1.0	
	Yes	1.1 (0.7–1.8)	
Mother tongue	Spanish	1.0	
	Quechua or Aymara	1.0 (0.5–2.1)	
Farmer	No	1.0	1.0
	Yes	1.9 (1.2–3.1)	1.7 (0.8–3.4)
Reading	No	1.0	
	Yes	1.6 (0.8–3.2)	
Education in years	Mean(SD)	1.0 (0.9–1.1)	
Wall material	Brick or concrete	1.0	1.0
	Wood and others	1.9 (1.1–3.3)	1.0 (0.4–2.7)
Drainage system	Septic tank	1.0	1.0
	Cesspool and others	2.3 (1.2–4.5)	2.0 (0.6–6.8)
Cooking fuel	Wood	1.0	
	Gas	1.5 (0.9–2.8)	
Number of rooms	3 or more	1.0	
	Fewer than 3	1.8 (1.1–2.9)	
Number of persons per room mean (SD)		1.21 (1.04–1.40)	1.2 (1.0–1.4)
Ownership of dogs	Yes	1.0	
	No	1.1 (0.7–2.0)	
Ownership of hens	No	1.0	1.0
	Yes	2.5 (1.1–5.9)	2.4 (0.9–6.1)
Use of bed net	Always	1.0	
	Sometimes	5.7 (0.7–47.6)	
Use of repellent	Always	4.9 (0.6–42.0)	
	Sometimes	1.0	
	Never	1.4 (0.7–2.8)	
House fumigations	Very often or always	1.2 (0.7–2.2)	
	Sometimes	1.0	
	Never	1.2 (0.7–2.1)	
Sleeping with windows open	Always	1.0	1.0
	Sometimes	2.2 (1.3–3.7)	3.2 (1.6–6.6)

## Discussion

This is the first study to assess both sociodemographic characteristics and housing conditions as risk factors for leishmaniasis in Bolivia. In our study, sex was the only one statistically significant variable for CL risk. This result is in agreement with the findings of the previous study of the risk factors carried out in 1997, where only sex and age were significant. These findings reflect of a mostly sylvatic transmission of CL in Bolivia where the absence of significance with the sociodemographic and housing condition variables could be interpreted as low peridomestic transmission for CL in the region. The importance of the variable sex is consistent when studies have been performed in areas where the chain transmission is sylvatic [15, 32, 33]. However, in research carried out in areas where transmission is peridomestic, no significant difference by sex has been found [34, 35].

Differences in risk for CL between men and women are related to differences in gender roles and not to biological characteristics associated with sex [36]. In fact, in sylvatic environments, leishmaniasis disease can be considered as a *lifestyle bodymark* [37] of men who have higher exposition to the transmitter vector of leishmaniasis. In these environments, the ideal male figure is characterized by being strong, courageous, a risk-taker, and able to withstand extensive physical sacrifices as well as dealing with danger [38]. For this reason, from a very young age, males are involved in recreational activities performed in the forest, such as hunting and fishing. One study conducted in Brazil showed that leisure activities in the forest in addition to fishing and hunting represented a higher risk of CL [13]. In addition, when males reach adulthood, their work activities—such as agriculture, gold mining, logging, and forestry—are also carried out inside the forest. One study performed in Ecuador supports this idea showing that the risk of CL was similar during adolescence but increased for men sharply into adulthood when they started work activities in the rainforest [9].

Regarding housing conditions, in our study, none of these factors were statistically significantly related to CL. Studies from Argentina and Ecuador have shown that the characteristics of the domicile are important determinants for the risk of becoming ill when the transmission chain is peridomestic [9, 11]. In this sense, it is possible to characterize vulnerable dwellings as those constructed with non-durable materials, which allow the formation of cracks through which the vectors can enter into houses. The lack of relevance of these factors in our study may be supportive of a sylvatic transmission chain.

The presence of animals in the house is also important when the transmission is peridomestic. In our study, the possession of hens was not statistically significant in the adjusted model. While some studies have suggested the certain role of hens in the transmission of leishmaniasis [39, 40], others have questioned the effect of avian blood on Leishmania development in sand flies [41].

No association between possession of dogs and CL risk was found either. The role of dogs in CL transmission is still discussed in the scientific literature. Some studies have suggested that the dog may act as a reservoir because a greater number of human cases were found in areas with high incidence of canine cases [42]. Other studies have shown the presence of parasitic DNA in the blood and bone marrow of these animals. However, these findings are considered to be circumstantial. Experimental studies showed that the vector was not infected when they were fed with blood from non-ulcerated areas of infected dogs. Another study showed that the percentage of infected vectors consuming blood from an infected dog was less than 1% [43]. Additionally, a review study on the possible role of a dog as a reservoir showed that there was no conclusive information to confirm this assumption after reviewing more than 90 studies [44].

Finally, the use of bed nets was not important in our study probably because most of the participants affirmed using them frequently to sleep. Studies from Colombia and Venezuela have shown that the use of bed nets only is not protective and that impregnation with insecticides such as deltamethrin [45] or lambdacyhalothrin [46] is important to reduce the risk of being bitten by the vector. Their utility seems to be very promising because of the long residual effect, with a residual lethal effect close to 100% after 12 months [47].

### Limitations

This study has three limitations related to the study design, the sample size, and the selection bias.

First, the cross-sectional study design does not allow us to discern causal relationships; however, this study does not aim to identify causal relations, but rather using statistical multivariate methods, it aims to test the effects of some new variables (sociodemographic and housing condition factors) as risk factors in the presence of other well-known risk factors such as sex, age, and occupation. Second, the small sample size is probably the most important limitation of the study. This limitation could be responsible for the lack of statistical significance of the variables included in the multivariate model. This limitation was mainly due to logistical difficulties related to the characteristics of the disease. On the one hand, CL occurs mainly in populations settled in sylvatic areas. Their communities are mostly small, scattered, and difficult to reach. On the other hand, their agricultural activities limited the participation of some of the inhabitants, since it was not possible to locate them in their houses during the day.

Third, given that the study did not use a random sampling method, a selection bias influenced by the willingness to participate in the study could have been possible. However, with the available data, the extent of this bias could not be assessed. Some variables such as the source of water, electricity, and wall material did not have enough variation in one of the communities which may have affected the analysis estimation.

## Conclusions

This is the first study performed in Bolivia on risk factors that consider sociodemographic and housing conditions in which sex was the only factor associated with CL. This finding suggests a sylvatic transmission mechanism in the study area. However, it is necessary to carry out larger studies in different locations of the country that will allow us to identify whether different mechanisms of transmission are present and in consequence whether the population is at risk. In addition, it is advisable to focus on CL education and prevention policies at an early age in sylvatic areas, with the aim of encouraging the use of protective measures that reduce vector exposure when people enter into the forest, for activities related to either leisure or work. This study contributes to the construction of knowledge on a little explored issue such as the risk factors in the sylvatic areas of the American Amazon.

## Abbreviations

ACL: American cutaneous leishmaniasis
CL: Cutaneous leishmaniasis
MST: Montenegro skin test
TIPNIS: Isiboro Secure National Park and Indigenous Territory
