# Correction to: risk factors for cutaneous leishmaniasis in the rainforest of Bolivia: a cross-sectional study

**DOI:** 10.1186/s41182-018-0103-z

**Published:** 2018-06-21

**Authors:** Daniel Eid, Miguel Guzman-Rivero, Ernesto Rojas, Isabel Goicolea, Anna-Karin Hurtig, Daniel Illanes, Miguel San Sebastian

**Affiliations:** 1 Institute of Biomedical Research, Faculty of Medicine, San Simon University, Aniceto Arce Avenue, 371 Cochabamba, Bolivia; 2 Department of Public Health and Clinical Medicine, Epidemiology and Global Health, Umea University, Umea, Sweden

## Correction

In the original publication of this article [1], the authors noted that some data were incorrect in Table 4. The corrected data are given in this erratum:

**Table 4:** Univariate and multivariate analysis of sociodemographic, housing conditions and protective activities associated with CL

Factor		Unadjusted OR with 95%CI	Adjusted OR with 95%CI
Sex	Female	1.0	1.0
	Male	2.2 (1.3–3.7)	3.2 (1.6–6.6)
Age in years	Mean(SD)	1.0 (1.0–1.0)	
Residence	Chipiriri community	1.0	1.0
	Ichoa community	1.9 (1.1–3.3)	1.9 (0.6–5.7)
Years of residence	More than 10	1.0	
	Below 10	1.3 (0.8–2.2)	
Migrant from high lands	No	1.0	
	Yes	1.1 (0.7–1.8)	
Mother tongue	Spanish	1.0	
	Quechua or Aymara	1.0 (0.5–2.1)	
Farmer	No	1.0	1.0
	Yes	1.9 (1.2–3.1)	1.7 (0.8–3.4)
Reading	No	1.0	
	Yes	1.6 (0.8–3.2)	
Education in years	Mean(SD)	1.0 (0.9–1.1)	
Wall material	Brick or concrete	1.0	1.0
	Wood and others	1.9 (1.1–3.3)	1.0 (0.4–2.7)
Drainage system	Septic tank	1.0	1.0
	Cesspool and others	2.3 (1.2–4.5)	2.0 (0.6–6.8)
Cooking fuel	Wood	1.0	
	Gas	1.5 (0.9–2.8)	
Number of rooms	3 or more	1.0	
	Fewer than 3	1.8 (1.1–2.9)	
Number of persons per room Mean(SD)		1.21 (1.04–1.40)	1.2 (1.0–1.4)
Ownership of dogs	Yes	1.0	
	No	1.1 (0.7–2.0)	
Ownership of hens	No	1.0	1.0
	Yes	2.5 (1.1–5.9)	2.4 (0.9–6.1)
Use of bed net	Always	1.0	
	Sometimes	0.7 (0.3–1.6)	
Use of repellent	Always	1.0	
	Sometimes	5.7(0.7–47.6)	
	Never	4.9(0.6–42.0)	
House fumigations	Very often or always	1.0	
	Some times	1.4(0.7–2.8)	
	Never	1.2 (0.7–2.2)	
Sleeping with windows open	Always	1.0	
	Sometimes	1.2 (0.7–2.1)	
